# 2440 Teaching rigor, reproducibility, and transparency using gamification

**DOI:** 10.1017/cts.2018.227

**Published:** 2018-11-21

**Authors:** James Willig, Jennifer Croker, Brian Wallace, David Dempsey, Brian Wallace, David Redden

**Affiliations:** University of Alabama at Birmingham

## Abstract

OBJECTIVES/SPECIFIC AIMS: The objectives for the Rigor, Reproducibility, and Transparency course within KAIZEN-Edu was to provide a platform that allows essential training, in a novel and customizable approach, for a large number of students across the multiple institutions within the UAB CCTS Partner Network. Successful implementation across this geographically diverse of partner institutions would serve as proof of concept to future dissemination across the CTSA consortium. METHODS/STUDY POPULATION: We used the “build a game” tools within Kaizen-Edu to design the “Rigor and Reproducibility Game.” The games consisted of four modules, with 20 questions designed to test participant knowledge, and edify learners on particular concepts through a multimedia approach (embedded video, text, and hyperlinks to articles) with content provided as questions released over 4 weeks. Researchers from across the UAB CCTS Partner Network developed comprehensive modules for (1) How Scientists Fool Themselves/Scientific Premise, (2) Authentication of Chemical and Biologic Resources and Sex and Other Biologic Variables, (3) Statistical Rigor, and (4) Comprehensive Review. A typical week began with review articles (1–2) sent to each participant. The participants are informed that 5 questions will be released midweek testing the key concepts from the papers. When ready, the participant logs into Kaizen-Edu and starts to answer questions/play the game. Immediately, the articles are opened for reference, followed by a brief 4–5 minute video which reinforces key concepts and then timed questions begin. A typical question is allowed 3 minutes (visible countdown clock). Accurate responses result in the addition of points, with double points awarded for correct answers within the questions time limit. No points are awarded for incorrect answers. After each question, a detailed explanation reviews and reinforces the key concepts. Each participants’ points contribute to both their individual score and team scores, which influences their position on the Rigor and Reproducibility game leaderboard. RESULTS/ANTICIPATED RESULTS: Within 2017, the Rigor Reproducibility, and Transparency course was conducted 5 times. A total of 126 researchers across 9 institutions were enrolled. A total of 87 enrollees completed the full course, with 80% passing (answering ≥75% of questions correctly) on their first attempt and an additional 20% passing on a second attempt. The distribution of completers across the CCTS Network was UAB=48, Auburn=13, Pennington=10, University of Alabama=5, Hudson Alpha=5, Tulane=4, University of South Alabama=1, LSU=2, and Southern Research=1. Researchers throughout at Partner Institutions represent 46% of the total population trained. DISCUSSION/SIGNIFICANCE OF IMPACT: This software based, gamification-enhanced course was broadly accepted with each session fully enrolled, and learners spread almost evenly between our institution and various Partner Network sites. Our pilot proved that gamification was an effective technique to engage users and produced a high pass rate, suggesting that the content both engaged learners and was effectively internalized. Educational interventions, imbued with principles of gamification provide educators powerful tools that use competition and/or collaboration to disseminate knowledge, engage learners with content, and save educator time as created game content can be reused in additional educational sessions. Analyses of the data trail provided by users engaging with such electronic learning tools will provide educators will insights on how to maximize learning, opening the door to an era of educational analytics.

